# Laquinimod dampens hyperactive cytokine production in Huntington's disease patient myeloid cells

**DOI:** 10.1111/jnc.13553

**Published:** 2016-04-05

**Authors:** Lucianne Dobson, Ulrike Träger, Ruth Farmer, Liat Hayardeny, Pippa Loupe, Michael R. Hayden, Sarah J. Tabrizi

**Affiliations:** ^1^ Department of Neurodegenerative DiseasesUniversity College LondonInstitute of Neurology and National Hospital for Neurology and NeurosurgeryLondonUK; ^2^ Now at German Cancer Research CentreImmune ToleranceTumour Immunology ProgramHeidelbergGermany; ^3^ Department of Medical StatisticsLondon School of Hygiene & Tropical MedicineLondonUK; ^4^ Teva PharmaceuticalsResearch and DevelopmentNetanyaIsrael

**Keywords:** cytokines, Huntington's disease, inflammation, laquinimod, neurodegenerative disease, NFκB

## Abstract

Huntington's disease (HD) is a neurodegenerative condition characterized by pathology in the brain and peripheral tissues. Hyperactivity of the innate immune system, due in part to NFκB pathway dysregulation, is an early and active component of HD. Evidence suggests targeting immune disruption may slow disease progression. Laquinimod is an orally active immunomodulator that down‐regulates proinflammatory cytokine production in peripheral blood mononuclear cells, and in the brain down‐regulates astrocytic and microglial activation by modulating NFκB signalling. Laquinimod had beneficial effects on inflammation, brain atrophy and disease progression in multiple sclerosis (MS) in two phase III clinical trials. This study investigated the effects of laquinimod on hyperactive proinflammatory cytokine release and NFκB signalling in HD patient myeloid cell cultures. Monocytes from manifest (manHD) and pre‐manifest (preHD) HD gene carriers and healthy volunteers (HV) were treated with laquinimod and stimulated with lipopolysaccharide. After 24 h pre‐treatment with 5 μM laquinimod, manHD monocytes released lower levels of IL‐1β, IL‐5, IL‐8, IL‐10, IL‐13 and TNFα in response to stimulation. PreHD monocytes released lower levels of IL‐8, IL‐10 and IL‐13, with no reduction observed in HV monocytes. The effects of laquinimod on dysfunctional NFκB signalling in HD was assessed by inhibitor of kappa B (IκB) degradation kinetics, nuclear translocation of NFκB and interactions between IκB kinase (IKK) and HTT, in HD myeloid cells. No differences were observed between laquinimod‐treated and untreated conditions. These results provide evidence that laquinimod dampens hyper‐reactive cytokine release from manHD and preHD monocytes, with a much reduced effect on HV monocytes.

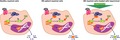

Evidence suggests targeting CNS and peripheral immune disruption may slow Huntington's disease (HD) neurodegenerative processes. The effects of laquinimod, an orally active immunomodulator, on hyperactive cytokine release and dysfunctional NFκB signalling in stimulated myeloid cell cultures from pre‐manifest and manifest HD gene carriers and healthy volunteers were investigated. Laquinimod dampened cytokine release but did not impact NFκB signalling.

Read the **Editorial Highlight** for this article on page 670.

**Abbreviations used**
(m)HTT: (mutant) huntingtin protein
AD: Alzheimer's disease
ASO: antisense oligonucleotide
Aβ: amyloid‐beta
BDNF: brain‐derived neurotrophic factor
BSA: bovine serum albumin
CAG: cytosine‐adenine‐guanine
CB_2_: cannabinoid receptor 2
CI: confidence Interval
CNS: central nervous system
CT: continued treatment
DIV: days *in vitro*
FBS: foetal bovine serum
GAPDH: glyceraldehyde 3‐phosphate dehydrogenase
GM‐CSF: granulocyte macrophage colony‐stimulating factor
HDAC: histone deacetylase
HD: huntington's disease
*HTT*: huntingtin gene
HV: healthy volunteers
IFNγ: interferon gamma
IKK: inhibitor of kappa B kinase
IL: interleukin
iNOS: inducible nitric oxide synthase
IκB: inhibitor of kappa B
LPS: lipopolysaccharide
MACS: magnetic‐activated cell sorting
manHD: manifest HD patients
MNC: mononuclear cell
MSD: MesoScale discovery
MS: multiple sclerosis
NFκB: nuclear factor kappa B
Nrf2: nuclear factor E2‐related factor 2
p.d.u.: procedure defined unit
PBMC: peripheral blood mononuclear cell
PE: phycoerythrin
PLA: proximity ligation assay
preHD: pre‐manifest Huntington's disease gene carriers
PT: pre‐treatment
RIPA: radioimmunoprecipitation assay
RPMI: roswell Park Memorial Institute
SDS: sodium dodecyl sulphate
TFC: total functional capacity
TGFβ: transforming growth factor beta
Th: T helper cell
TLR: toll‐like receptor
TNFα: tumour necrosis factor alpha
UCL: University College London
WGA: wheat germ agglutinin

Huntington's disease (HD) is a neurodegenerative condition caused by a cytosine‐adenine‐guanine (CAG) repeat expansion in exon 1 of the huntingtin gene (*HTT*) (The Huntington's Disease Collaborative Research Group 1993) and is characterized by progressive motor, cognitive and psychiatric symptoms including involuntary movement disturbances, dementia and depression. Symptoms display delayed onset despite presence of mHTT throughout the entire lifetime, and usually become manifest in middle age. Primary pathology involves dysfunction and loss of the GABAergic medium spiny neurons in the striatum as well as cortical neuronal degeneration, and is hallmarked by presence of intra‐nuclear and cytoplasmic aggregates of mHTT. Many mechanisms have been described to contribute to tissue damage leading to neuronal degeneration in HD, including excitotoxicity, mitochondrial dysfunction, production of free radicals, impairment of protein degradation systems and transcriptional dysregulation; leading to inflammatory processes that cause microglial and astrocytic activation and neuronal loss (Ross and Tabrizi 2011; Ellrichmann *et al*. 2013).

In the central nervous system (CNS), elevated levels of inflammatory molecules are observed in postmortem HD brain tissue (Björkqvist *et al*. 2008). Substantial numbers of activated microglia are seen in affected regions of the brain such as the striatum and cortex (Sapp *et al*. 2001; Pavese *et al*. 2006), and this correlates with loss of neuronal function (Politis *et al*. 2011). Activated microglia are also present in the brains of pre‐symptomatic HD gene carriers (Tai *et al*. 2007), and peripherally, inflammatory molecules are elevated in blood plasma many years before disease onset (Dalrymple *et al*. 2007; Björkqvist *et al*. 2008; Wild *et al*. 2011), suggesting a possible active role of the innate immune system during progression to symptomatic disease. Peripheral myeloid cells are the likely source of elevated systemic levels of inflammatory molecules. HD patient monocytes and macrophages produce increased levels of cytokines when stimulated *ex vivo* (Björkqvist *et al*. 2008), and are impaired in their ability to migrate towards chemo‐attractant stimuli (Kwan *et al*. 2012b). These same dysfunctional phenotypes are observed in both peripheral myeloid cells and microglia isolated from HD mouse models (Björkqvist *et al*. 2008), suggesting that blood‐derived myeloid cells may reflect the pathological actions of microglia in the CNS in HD. It is also possible that there is direct communication between the peripheral immune system and the CNS in HD: peripheral inhibition of kynurenine 3‐monooxygenase, highly expressed in peripheral immune cells, ameliorates neurodegeneration in a HD mouse model by raising CNS levels of neuroprotective metabolite kynurenic acid and preventing hyperactive microglial activity (Zwilling *et al*. 2011).

Hyper‐reactivity of the innate immune response in HD is due partly to nuclear factor (NF)κB pathway dysregulation (Khoshnan *et al*. 2004; Träger *et al*. 2014). In HD myeloid cells, dysregulation is observed upon activation of Toll‐like receptors (TLRs) using lipopolysaccharide (LPS), implicating a possible mechanism of dysfunctional signalling downstream of TLR4, the principal LPS receptor (Poltorak *et al*. 1998; Qureshi *et al*. 1999). In cultured cells expressing mHTT and striatal cells from HD transgenic mice, mHTT was found to interact with inhibitor of kappa B (IκB) kinase (IKK) γ subunit, leading to elevated NFκB activity and increased NFκB‐dependent gene expression (Khoshnan *et al*. 2004). This was also observed in primary human peripheral blood mononuclear cells (PBMCs), along with a more rapid degradation of IκB following LPS stimulation in HD monocytes compared to controls (Träger *et al*. 2014).

While there are treatments that manage the severity of symptoms in HD, there are no effective disease‐modifying therapies and consequently, novel approaches targeting hyperactive immune cells have been investigated. Minocycline, a caspase inhibitor, reduces IL‐1β and inducible nitric oxide synthase, and in the R6/2 mouse model of HD, minocycline significantly delayed disease progression (Chen *et al*. 2000). A small study in HD patients has shown minocycline to be safe and well tolerated (Thomas *et al*. 2004), however, the primary endpoint of a 25% improvement in the total functional capacity score was not met in a subsequent futility study (Huntington Study Group DOMINO Investigators 2010). More recently, dimethyl fumarate, an immunomodulator approved as a treatment for relapsing‐remitting MS (Tecfidera, Biogen), has been shown to have beneficial effects on neuronal degeneration, motor functions and survival time in R6/2 and YAC128 mouse models of HD. Dimethyl fumarate is believed to exert its effects via induction of nuclear factor E2‐related factor 2, a transcription factor which activates detoxification pathways and subsequently protects against oxidative damage triggered by inflammation (Ellrichmann *et al*. 2011).

Laquinimod is a novel oral immunomodulatory drug which has been shown to reduce the proportion of astrocytes with nuclear NFκB (p65/RelA) immune‐reactivity in cuprizone‐treated mice (Brück *et al*. 2012), and decrease cytokine release from LPS‐stimulated adult human microglia (Mishra *et al*. 2014). Laquinimod effects on peripheral inflammatory phenotypes have also been described. Laquinimod‐treated human PBMCs release lower levels of interleukin (IL)‐17, IL‐3 and granulocyte colony‐stimulating factor (Brück and Wegner 2011). Mononuclear cells isolated from the spleen of a laquinimod‐treated MS rat model display evidence of a shift from proinflammatory T helper (Th)1‐activating to anti‐inflammatory and regulatory Th2/Th3‐activating cytokine expression profile, with a laquinimod‐induced change in favour of the Th2 stimuli IL‐4, IL‐10 and transforming growth factor beta (TGFβ) and reductions in the Th1 stimuli tumour necrosis factor alpha (TNFα) and IL‐12 (Yang *et al*. 2004). Laquinimod has also been shown to promote splenic development of M2 polarized monocytes and dendritic cells in mice, leading to reduced cellular production of Th1‐activating cytokines IL‐6, IL‐12 and TNFα with a corresponding increase in Th2‐activating anti‐inflammatory IL‐10 production (Schulze‐Topphoff *et al*. 2012).

Affymetrix GeneChip arrays on PBMCs have shown that laquinimod increases IκB expression whilst downstream NFκB genes are decreased (Gurevich *et al*. 2010), and imaging flow cytometry has shown laquinimod‐induced reduction in p65 translocation in primary murine astrocytes (Brück *et al*. 2012), suggesting a potential role of NFκB signalling in laquinimod mechanism of action. It is therefore plausible that laquinimod could reverse the hyperactive proinflammatory phenotype observed in HD PBMCs, possibly by rescuing the dysfunctional NFκB signalling in these cells. Intervention of peripheral inflammatory events has previously been shown to modulate central neuropathology and disease progression in HD (Zwilling *et al*. 2011; Bouchard *et al*. 2012; Kwan *et al*. 2012a), therefore an immunomodulatory drug such as laquinimod is a potential candidate for slowing disease progression in HD patients by affecting peripheral immune cells. In addition, laquinimod has already been studied in human immunomodulation and has a very good safety profile following clinical trials for the treatment of MS (Comi *et al*. 2012; Filippi *et al*. 2014; Vollmer *et al*. 2014). Such studies have also provided evidence for laquinimod‐induced immunomodulation having beneficial effects on disease progression and brain atrophy in MS (Comi *et al*. 2012). As inflammation has been implicated in myelin, axonal and neuronal loss in HD pathology (Ellrichmann *et al*. 2013), it is feasible that laquinimod may slow disease progression and reduce brain atrophy rate in HD through central and peripheral immunomodulatory mechanisms, as it has been shown to do in MS.

Therefore, the goal of this study was to determine whether laquinimod down‐regulates the hyper‐reactive proinflammatory phenotype of HD patient myeloid cells, and switches the functional signatures of these cells away from M1 and towards M2 polarization with a consequential shift in the balance from Th1‐ to Th2‐activating cytokine release. This is the first demonstration of the effects of laquinimod in HD patient myeloid cells. The second aim was to elucidate a possible mechanism of action of laquinimod in the periphery, by assessing its possible effects on NFκB pathway dysregulation in HD patient myeloid cells. While a mechanism of action involving the NFκB pathway has been shown in cells of the CNS, it is not yet known whether laquinimod has the same effect in the periphery. To this end, primary human monocyte and macrophage cultures from HD patient subgroups and healthy volunteers were treated with laquinimod in stimulated and non‐stimulated conditions. Cytokine release, IκB levels, NFκB translocation and IKKγ‐mHTT interactions were examined.

## Methods

### Collection and classification of human samples

All human experiments were performed in accordance with the Declaration of Helsinki and approved by University College London (UCL)/UCL Hospitals Joint Research Ethics Committee. All blood donors provided informed written consent for research on Huntington's disease. Whole blood samples were collected from manifest HD patients with early or moderate stage disease (manHD), pre‐manifest HD gene carriers (preHD) and healthy volunteers (HV). ManHD samples were collected from six females and 13 males with an average age of 55.32 years ranging from 27 to 70; preHD samples were collected from six females and three males with an average age of 47.45 years ranging from 33 to 62; HV samples were collected from 10 females and five males with an average age of 47.95 years ranging from 25 to 68.

### Primary human monocyte isolation

Whole blood samples were decanted into 50 mL heparinized tubes (50 μL prescription grade heparin in standard 50 mL Falcon tubes). 25 mL whole blood was then carefully layered onto 20 mL Histopaque‐1077 (Sigma, Dorset, UK) and separated through the gradient by centrifugation in a swinging bucket rotor at 400 *g* for 30 min at 21°C with no brake. The resulting PBMC layer was removed with a Pasteur pipette from the plasma/Histopaque‐1077 interface. PBMCs were washed in magnetic‐activated cell sorting (MACS) buffer [1 × Dulbecco's phosphate‐buffered saline (Gibco, Life Technologies, Paisley, UK); 0.5% bovine serum albumin (Sigma); 2 mM EDTA (Sigma)] and pelleted out of wash solution by centrifugation at 350 *g* for 10 min at 4°C. Cells were re‐suspended in 1 mL MACS buffer and 60 μL anti‐CD14 MACS MicroBeads (Miltenyi Biotec, Surrey, UK) was added to the suspension, followed by quick vortex and 15 min incubation at 4°C. Cells were then pelleted by centrifugation at 350 *g* for 5 min at 4°C, and re‐suspended in fresh MACS buffer before being applied to MACS columns (Miltenyi Biotec) mounted on a magnetic separator (Miltenyi Biotec). The flow‐through was discarded and magnetically isolated CD14 + monocytes were plunged out of the columns into separate collection tubes.

### Cell culture

Primary human monocytes were seeded in Primaria culture dishes (BD Falcon, BD Biosciences, Oxford, UK) at 3 × 10^5^ cells/cm^2^, and maintained in Roswell Park Memorial Institute medium 1640 (Gibco, Life Technologies); 10% foetal bovine serum (Gibco); 2 mM L‐glutamine (Gibco, Life Technologies); 5 U/mL penicillin and 5 μg/mL streptomycin (Gibco PenStrep solution) in 5% CO_2_ atmosphere at 37°C. Pre‐testing has shown that this protocol produces monocyte cultures of at least 95% CD14^+^ monocytes and these were used in experiments from 1 to 3 days *in vitro* (DIV). For some experiments, monocytes were differentiated into macrophages with the addition of 20 ng/mL granulocyte macrophage colony‐stimulating factor (GM‐CSF, R&D Systems, Oxford, UK) to the culture medium on seeding. Cells were given a complete media change with addition of fresh GM‐CSF at 3 DIV and have been confirmed by factor analysis to be fully differentiated macrophages at 6 DIV and were used in experiments at this stage.

### Cell treatments

Once the cells had adhered post‐seeding, monocytes were treated with doses of laquinimod ranging from 0 to 100 μM for 24 or 48 h for toxicity analysis. For cytokine analysis, monocytes were treated with 1 or 5 μM laquinimod for 2 or 24 h, or were untreated. Cells were then given a media change, and relevant cultures were stimulated with 1 μg/mL LPS (Sigma, #L6529) for 24 h. 10 ng/mL interferon gamma (IFNγ, R&D Systems) was added at the same time as LPS as a priming agent. Three drug treatment durations, and two drug concentrations were used: 2 and 24 h pre‐treatments of 1 and 5 μM laquinimod (before stimulation, 2hPT and 24hPT respectively) and 24 h 5 μM pre‐treatment + 24 h continued 5 μM laquinimod treatment during LPS stimulation (24hPT+24hCT). After LPS stimulation, culture supernatants were collected, frozen and stored at −80°C. For western blot analysis, monocytes received 24hPT with 5 μM laquinimod, or untreated, followed by 1 μg/mL LPS stimulation over a time‐course of 0–60 min. For imaging flow cytometry analysis, monocytes received 24hPT with 5 μM laquinimod, or untreated, before stimulation with 1 μg/mL LPS for 45 min. For proximity ligation assays, macrophages were treated with 5 μM laquinimod for 24 h, and then fixed.

### Cytotoxicity assay

Monocytes cultures from six HD patients and HV were treated, in duplicate, with 0, 0.001, 0.01, 0.1, 0.5, 1, 5, 10, 50 or 100 μM laquinimod for 24 or 48 h. Cell survival after laquinimod treatment was assessed by CytoTox 96 Non‐Radioactive Cytotoxicity Assay (Promega, Southampton, UK), as per the manufacturer's protocol, and compared to viability of untreated cells.

### ELISA analysis of cytokines

Cytokine levels in monocyte culture supernatants were measured using MesoScale Discovery Human Th1/Th2 10‐Plex Tissue Culture Kit analysing 10 different cytokines simultaneously (IFNγ, IL‐1β, IL‐2, IL‐4, IL‐5, IL‐8, IL‐10, IL‐12p70, IL‐13, TNFα), or Human IL‐6 Singleplex Kit, according to the manufacturer's protocol. Depending on cell treatment condition, for 10‐plex analysis *n* = 9–11 for HV, *n* = 9 for preHD and *n* = 10–14 for manHD, and for the IL‐6 singleplex analysis *n* = 13–15 for HV, *n* = 6–9 for preHD and *n* = 15–19 for manHD. Cytokine level read‐outs were normalized to protein content per culture well, which was ascertained by lysing the cultures with radioimmunoprecipitation assay buffer [consisting of 25 mM Tris‐HCl pH 7.6, 150 mM NaCl, 1% Nonidet‐P40, 1% sodium deoxycholate, 0.1% sodium dodecyl sulphate and protease inhibitor cocktail (Roche, West Sussex, UK)] and performing bicinchoninic acid protein assays (Pierce, Life Technologies) on the resulting lysates, according to the manufacturer's protocol. IFNγ levels were not considered because of the addition of this cytokine as a priming agent in the stimulated conditions.

### Western blotting

Monocyte cultures from four manHD received 24hPT with 5 μM laquinimod, or untreated, followed by LPS stimulation. Cell lysates (made using radioimmunoprecipitation assay buffer) were harvested at 0, 5, 15, 30 and 60 min time‐points after stimulation. Protein lysates were analysed by western blot using anti‐IκB antibody (1:500, Santa Cruz Biotechnology, Heidelberg, Germany) to determine the rate of IκB degradation. All IκB readings were normalized to the house‐keeping protein glyceraldehyde 3‐phosphate dehydrogenase (anti‐GAPDH antibody, 1:3000, Sigma Aldrich, Dorset, UK), and quantified by densitometry analysis of three exposures using TotalLab TL100 image analysis software (Sigma Aldrich). Western blotting and analysis were performed as previously described in detail (Träger *et al*. 2014).

### Imaging flow cytometry

Monocyte cultures from seven manHD received 24hPT with 5 μM laquinimod, or untreated, followed by 45 min LPS stimulation. Monocytes were fixed and probed with NFκB p65 XP antibody (1:200, Cell Signaling Technology, New England Biolabs, Hertfordshire, UK) followed by secondary anti‐rabbit IgG phycoerythrin‐conjugated antibody (1 : 100, eBioscience Ltd., Hatfield, UK). Cells were stained in 1 μg/mL 4′,6‐diamidino‐2‐phenylindole (DAPI, Sigma Aldrich) nuclear stain immediately prior to analysis. Samples were run on ImageStreamX analyser (Amnis Corporation, Seattle, WA, USA) to assess intra/extra‐nuclear localization of p65 and analysed using IDEAS Software (Amnis) to calculate percentage translocated cells. Imaging flow cytometry and analysis of images were performed as previously described in detail (Träger *et al*. 2014).

### Proximity ligation assay

Monocytes from six manHD and preHD were seeded on 13 mm coverslips at 5 × 10^3^ cells/cm^2^, and differentiated into macrophages. Cells were treated with 5 μM laquinimod for 24 h, or untreated, then fixed in 4% paraformaldehyde and stained with fluorescent wheat germ agglutinin (WGA, Life Technologies) to label membranes. Cells were stained with 1 μg/mL WGA for 5 min at 37°C before washing ten times with phosphate‐buffered saline. Cells were then permeabilized, blocked and probed with mouse anti‐HTT antibody 4C9 (1 : 300, gift from Novartis, Basel, Switzerland) and anti‐IKKγ antibody (1 : 100, Santa Cruz). Instead of using fluorescently labelled secondary antibodies, a proximity ligation approach was undertaken, as per the manufacturer's instructions [Proximity Ligation Assay (PLA), Sigma]. Briefly, samples were incubated with secondary antibodies conjugated with PLUS and MINUS DNA probes, which were hybridized and ligated before amplification of the DNA template in a rolling circle amplification reaction. Detection solution was added to identify amplified DNA, and coverslips were mounted on glass slides in Duolink mounting medium (Sigma Aldrich) containing 1 μg/mL DAPI. Signals were detected using fluorescent confocal microscopy. Positive interactions appear as fluorescent spots which were quantified as average number of spots per cell using Volocity software (PerkinElmer, Waltham, MA, USA). Each of the two negative controls was made up of a single primary antibody with both PLUS and MINUS secondary antibodies. The positive control was anti‐IKKβ (1 : 50; Santa Cruz) and anti‐IKKγ primary antibodies with both secondary antibodies, as these proteins are known to directly interact. PLA and analysis of images were performed as previously described in detail (Träger *et al*. 2014).

### Statistical analyses

All samples were labelled with an anonymized 5‐digit number for blinded analysis

Cytotoxicity assay data were statistically analysed in‐house. One‐way ANOVA with Dunnett's multiple comparison tests comparing all laquinimod treatment conditions to untreated were used to analyse statistically significant variations in the means.

Analysis of ELISA cytokine measurements was performed by an independent statistician (RF). The data set was split into two – stimulated and non‐stimulated conditions – to perform statistical analysis. Not every subject had a measurement for every condition because of some samples failing quality control, leading to unbalanced data. Data were log transformed prior to analysis to improve normality assumptions. A linear mixed model was fitted to each of the subsets separately, assuming exchangeable correlation, with robust standard errors to allow for misspecification of the covariance structure. Comparisons of interest were calculated using linear contrasts. This approach allows for data from a subject to be used even if some of the conditions are missing, under a missing at random assumption. For the HD combined comparisons, a weighted combination of preHD and manHD was used, based on the total number of patients in those subgroups. All analyses were additionally adjusted for age. Multiple comparison adjustments were not made because of the small sample size and lack of independency in cytokine activity, and this was taken into consideration when reviewing the findings from these analyses. All results of cytokine levels and statistical analysis of this data are graphically presented and reported on the logarithmic scale. It is important to note that natural differences in cytokine release from primary human monocytes leads to highly variable data, and the sample size limits the precision for which estimates of between condition and between group differences can be made. Therefore, in cases where there is absence of statistical evidence it is not possible to determine whether this is because of no effect or that the random variation in measurements masks any effect that may be present.

NFκB mechanism of action data was analysed in‐house. IκB degradation kinetics data were analysed using two‐way repeated measures ANOVA comparing treatment conditions (untreated or laquinimod pre‐treatment) over time post‐LPS‐stimulation. Two‐tailed, paired Student's *t*‐tests were used to compare untreated versus laquinimod treatments for imaging flow cytometry and PLA data. For PLA analysis, all conditions were tested in six subjects, however, the results for the laquinimod condition were removed for one of the subjects for quality control reasons.

## Results

### Laquinimod is not toxic to *ex vivo* human monocyte cultures

**Figure 1:**
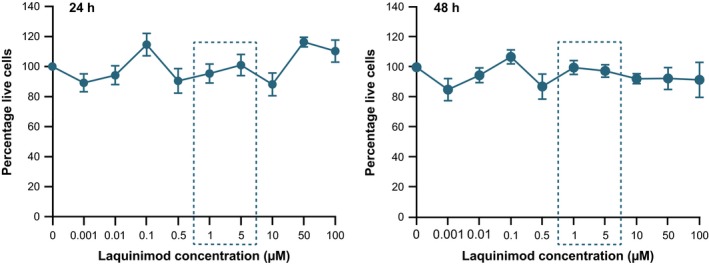
Primary human monocyte survival following laquinimod treatment. Monocytes treated with a range of laquinimod concentrations for 24 or 48 h. Laquinimod‐treated monocyte survival expressed as a percentage of untreated monocyte survival. Data points = mean average of six biological repeats. Error bars = SEM. Concentrations of 1 and 5 μM (boxed) were used in subsequent experiments.

First, it was important to determine whether laquinimod is toxic to primary human monocytes in culture. Monocytes were treated for 24 or 48 h with previously published doses of laquinimod used to treat human PBMCs, 1 and 5 μM (Brück and Wegner 2011), as well as a range of concentrations either side. Laquinimod‐treated cell survival was assessed relative to untreated cells. None of the laquinimod concentrations or treatment durations tested showed any overt toxicity to the cells (Fig. 1). Confidence intervals (CIs, calculated at the 95% confidence level) around the mean percentage survival relative to untreated cells span 100% survival in all laquinimod‐treated conditions, and are generally narrow. In addition, none of the laquinimod treatment conditions showed a statistically significant difference in percentage monocyte survival (*p* > 0.05). After the longest laquinimod treatment duration of 48 h, 1 and 5 μM laquinimod resulted in an average of 99.84% and 97.54% monocyte survival, respectively, relative to untreated cells. The lower bound of the CIs for these results were 88.01% for 1 μM laquinimod and 86.84% for 5 μM laquinimod, therefore one can be confident that at these concentrations the true cell survival relative to untreated is above 86%, which was taken to be acceptable in terms of establishing a lack of laquinimod toxicity. Previously published doses of 1 and 5 μM laquinimod were therefore used for subsequent experiments, which is in accordance with physiological *in vivo* data for relevant plasma levels of laquinimod in humans (Sennbro *et al*. 2006; Preiningerova 2009) and mice (Brunmark *et al*. 2002).

### Laquinimod reduces hyper‐reactive cytokine release from stimulated HD patient monocytes

HD monocytes and macrophages have been observed to release elevated levels of the proinflammatory Th1‐activating cytokines IL‐6, IL‐8 and TNFα (Björkqvist *et al*. 2008), and laquinimod has been reported to drive a shift from Th1‐ to Th2‐activating cytokine production in human PBMCs *in vitro* (Brück and Wegner 2011) and in mouse and rat models of MS *in vivo* (Zou *et al*. 2002; Yang *et al*. 2004; Brück and Wegner 2011). To determine whether laquinimod can shift the hyper‐reactive proinflammatory cytokine release from HD myeloid cells by tipping the reactive cytokine balance from Th1‐ to Th2‐activating cytokine release, *ex vivo* primary monocyte cultures from HV, preHD and manHD were treated with laquinimod, or untreated, in non‐stimulated and stimulated conditions. Culture supernatants were collected and production of the following cytokines was measured: Th1‐activating cytokines IL‐1β, IL‐6, IL‐8, IL‐12p70, TNFα; Th2‐activating cytokines IL‐4, IL‐5, IL‐10, IL‐13, TNFα; and IL‐2.

#### Baseline cytokine release in untreated monocytes

Levels of all cytokines, other than IL‐6, were significantly higher (*p* < 0.05), or borderline, in manHD compared to HV in monocyte cultures which had received no laquinimod treatment or stimulation. IL‐5, IL‐8 and IL‐13 were also elevated in preHD compared to HV under these conditions. While not all cytokines tested were higher in preHD compared to HV, both preHD and manHD subgroups showed similar trends, and combining the HD subjects increased the power sufficiently to see a statistically significant (*p* < 0.05), or borderline increase in all cytokines tested in HD compared to HV cultures (data not shown) suggesting that HD monocytes inherently release higher levels of cytokines.

#### Cytokine release in laquinimod‐treated non‐stimulated monocytes

Monocytes from HV showed at least borderline significant evidence for an increase in Th1‐activating cytokine IL‐12 (*p* = 0.062) (Figure S1) and the Th2‐activating cytokines IL‐4 (*p* = 0.009) and IL‐5 (*p* = 0.002) (Figure S2), after 2hPT with 1 μM laquinimod. After 24hPT with 1 μM laquinimod, IL‐4 and IL‐5 levels were still estimated to be higher, but to a lesser degree which was not statistically significant. When HV monocytes were treated with 5 μM laquinimod, there was evidence of an increase in levels of the Th1‐activating cytokines IL‐12 and TNFα (Figure S1) compared to untreated: IL‐12 was increased after 2hPT and 24hPT+24hCT (*p* = 0.036 and *p* = 0.026 respectively), and TNFα was increased after 24hPT and 24hPT+24hCT (*p* = 0.022 and *p* = 0.006 respectively). IL‐2 and the Th2‐activating cytokines IL‐4, IL‐5, IL‐10 and IL‐13 were also increased (Figure S2): IL‐2 was increased after 24hPT+24hCT (*p* = 0.030), IL‐4 was increased after 24hPT (*p* = 0.014), IL‐5 was increased after 2hPT, 24hPT and 24hPT+24hCT (*p* = 0.025, *p* = 0.002 and *p* = 0.007 respectively), IL‐10 was increased after 24hPT+24hCT (*p* = 0.004) and IL‐13 was increased after 24hPT+24hCT (*p* = 0.005). There was no evidence of a statistically significant effect of laquinimod treatment at any of the concentrations or treatment durations on non‐stimulated preHD or manHD monocytes.

#### Cytokine release in non‐laquinimod‐treated stimulated monocytes

In LPS(+IFNγ)‐stimulated conditions, there was some evidence of an increase in IL‐1β, IL‐5 and IL‐13 release in manHD compared to HV monocytes. The log levels of these cytokines in manHD were all estimated to be around 0.4 higher than in HV, with these differences being of at least borderline statistical significance. IL‐4, IL‐6 and IL‐10 had differences in similar magnitude but did not reach borderline significance. IL‐8 levels were lower in preHD compared to HV, with borderline statistical significance. Overall there was little statistical evidence of differences between HD subgroups and HV monocyte cytokine release in stimulated conditions (data not shown).

#### Cytokine release in 1 μM laquinimod‐treated stimulated monocytes

**Figure 2:**
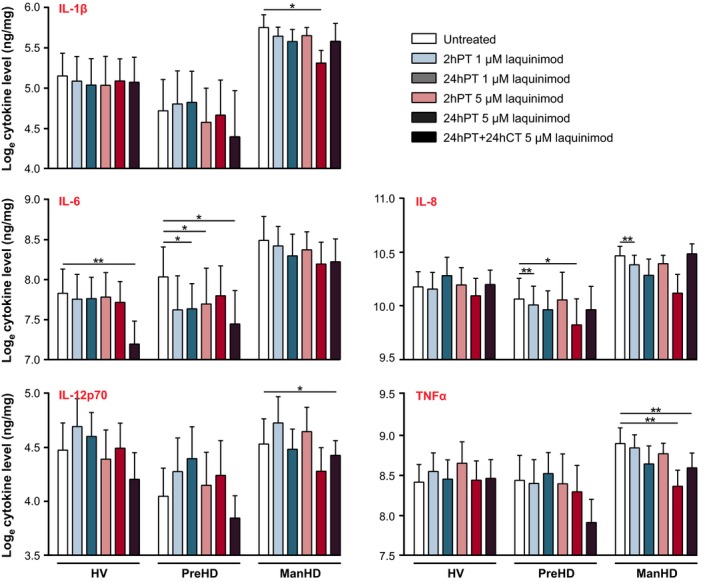
Th1‐activating cytokine release in stimulated monocytes pre‐treated with laquinimod. *Primary* stimulated human monocyte cultures from HV, preHD and manHD received 2hPT or 24hPT with 1 or 5 μM laquinimod, or 24hPT+24hCT with 5 μM laquinimod. Levels of cytokine release were measured in monocyte culture supernatants and normalized to cell culture protein. Figure 2 displays results for the Th1‐activating cytokines and Fig. 3 displays results for IL‐2 and the Th2‐activating cytokines measured. Columns = mean average log cytokine level of biological repeats; error bars = SEM; *p < 0.05, **p < 0.01.

**Figure 3:**
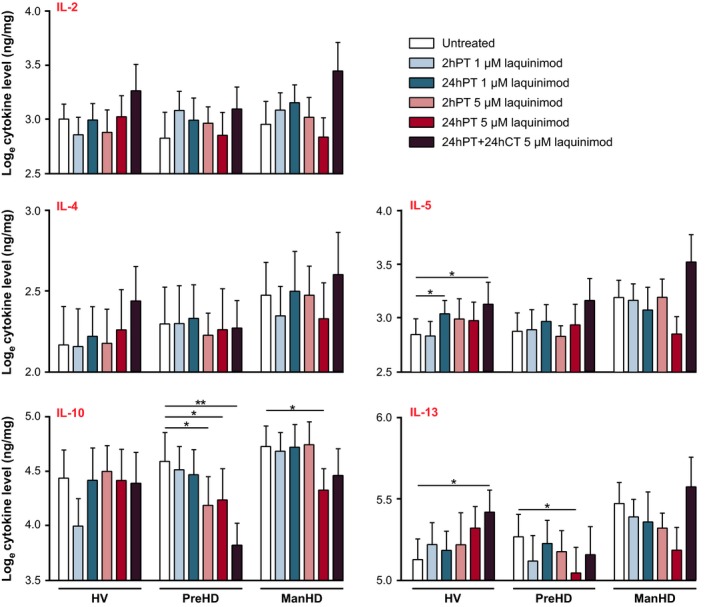
IL‐2 and Th2‐activating cytokine release in stimulated monocytes pre‐treated with laquinimod. Primary stimulated human monocyte cultures from HV, preHD and manHD received 2hPT or 24hPT with 1 or 5 μM laquinimod, or 24hPT+24hCT with 5 μM laquinimod. Levels of cytokine release were measured in monocyte culture supernatants and normalized to cell culture protein. Figure 2 displays results for the Th1‐activating cytokines and Figure 3 displays results for IL‐2 and the Th2‐activating cytokines measured. Columns = mean average log cytokine level of biological repeats; error bars = SEM; *p < 0.05, **p < 0.01.

There was no evidence of an effect of 2hPT with 1 μM laquinimod in LPS(+IFNγ)‐stimulated HV monocytes. Following 24hPT at the same concentration, HV monocytes showed a borderline significant increase in Th1‐activating cytokine IL‐8 (*p* = 0.064; Fig. 2) and a significant increase in Th2‐activating cytokine IL‐5 (*p* = 0.035; Figure 3) compared to untreated cells. PreHD subjects showed decreases in Th1‐activating cytokines IL‐6 and IL‐8 (*p* = 0.069 and *p* = 0.004 respectively; Figure 2) compared to untreated, following 2hPT with 1 μM laquinimod. After a longer pre‐treatment duration, 24hPT, at the same concentration, IL‐6 levels were significantly decreased compared to untreated (*p* = 0.028; Figure 2). Like the preHD group, manHD monocytes also showed a significant decrease in IL‐8 levels following 2hPT with 1 μM laquinimod in stimulated cells (*p* = 0.005; Figure 2). There was no statistical evidence of an effect of 1 μM laquinimod on stimulated manHD monocyte cytokine release following 24hPT (Figures 2 and 3).

#### Cytokine release in 5 μM laquinimod‐treated stimulated monocytes

In HV monocytes, 2hPT or 24hPT with the higher laquinimod concentration of 5 μM resulted in no significant effects on levels of cytokine release (*p* > 0.05 for all comparisons; Figures 2 and 3). Levels of Th1‐activating cytokine IL‐6 were significantly lower in HV monocytes which received 24hPT+24hCT with 5 μM laquinimod compared to untreated (*p* = 0.004; Figure 2), and levels of Th2‐activating cytokines IL‐5 and IL‐13 were significantly higher in the same condition (*p* = 0.047 and *p* = 0.041 respectively; Fig. 3).

In the preHD group, although not always statistically significant, the estimates comparing treatments for 2hPT, 24hPT and 24hPT+24CT with the untreated condition generally showed decreases in levels of cytokines. The exceptions were IL‐2 and IL‐5, where the general trend shows increases in cytokine levels following laquinimod treatments, but these differences were small in magnitude. Th1‐activating cytokines IL‐6 and IL‐8 were reduced (Fig. 2): IL‐6 was reduced after 2hPT and 24hPT+24hCT (*p* = 0.015 and *p* = 0.016 respectively), and IL‐8 was reduced after 24hPT (*p* = 0.015). Th2‐activating cytokines IL‐10 and IL‐13 were also reduced (Fig. 3): IL‐10 was reduced after 2hPT, 24hPT and 24hPT+24hCT (*p* = 0.037, *p* = 0.015 and *p* = 0.004 respectively), and IL‐13 was reduced after 24hPT (*p* = 0.033).

In manHD monocytes, 2hPT with 5 μM laquinimod did not show a statistically significant change in cytokine levels compared to untreated, however, the 24hPT effect was of at least borderline statistical significance for several cytokines, with evidence of a reduction in the proinflammatory, Th1‐activating cytokines IL‐1β (*p* = 0.022), IL‐6 (*p* = 0.056), IL‐8 (*p* = 0.052) and TNFα (*p* = 0.007) (Fig. 2), and the Th2‐activating cytokines IL‐5 (*p* = 0.053), IL‐10 (*p* = 0.044) and IL‐13 (*p* = 0.052) (Fig. 3). In addition, for the cytokines IL‐1β, IL‐10 and TNFα, 24hPT with 5 μM laquinimod resulted in a statistically significant reduction in cytokine release from manHD monocytes that was not seen in HV. While not statistically significant, many of the other cytokines also showed this trend. This could suggest that 24hPT with 5 μM laquinimod has more of an effect on reducing cytokine production in manHD monocytes than in HV monocytes. After 24hPT+24hCT, there were decreases in the Th1‐activating cytokines IL‐6 (*p* = 0.053), IL‐12 (*p* = 0.028) and TNFα (*p* = 0.009) compared to untreated in manHD monocytes (Fig. 2). Although not statistically significant, levels of IL‐2 and Th2‐activating cytokines IL‐4, IL‐5 and IL‐13 were increased following 24hPT+24hCT (Fig. 3), going against the trend seen with 2hPT and 24hPT.

A secondary analysis was performed to increase power and look at the differences between HV and HD overall. When manHD and preHD subgroup data were combined, 24hPT with 5 μM laquinimod resulted in at least a borderline significant decrease in the Th1‐activating cytokines IL‐1β (*p* = 0.006), IL‐6 (*p* = 0.059), IL‐8 (*p* = 0.005) and TNFα (*p* = 0.003), and the Th2‐activating cytokines IL‐10 (*p* = 0.003) and IL‐13 (*p* = 0.009) compared to the untreated condition. In addition, the difference in change from the untreated condition between grouped HD subjects and HV was of at least borderline significance for IL‐1β (*p* = 0.062), IL‐5 (*p* = 0.045), IL‐10 (*p* = 0.008), IL‐13 (*p* = 0.005) and TNFα (*p* = 0.045), suggesting that 24hPT with 5 μM laquinimod has a larger effect on reducing cytokine release from HD cells than HV cells.

### Laquinimod does not affect NFκB signalling in HD monocytes

Laquinimod‐induced down‐regulation of proinflammatory factors in monocytes has previously been shown to be because of a switch from MI to M2 phenotype (Zou *et al*. 2002; Yang *et al*. 2004; Brück and Wegner 2011; Schulze‐Topphoff *et al*. 2012). The exact mechanism for this remains unclear, although in astrocytes the down‐regulation has been shown to be a result of reduced NFκB activation (Brück *et al*. 2012). In HD myeloid cells, the enhanced release of proinflammatory factors is at least in part because of elevated NFκB activation (Träger *et al*. 2014), so investigations were made into a role of the NFκB pathway in the observed overall dampening of cytokine release from stimulated HD monocytes pre‐treated with laquinimod.

**Figure 4:**
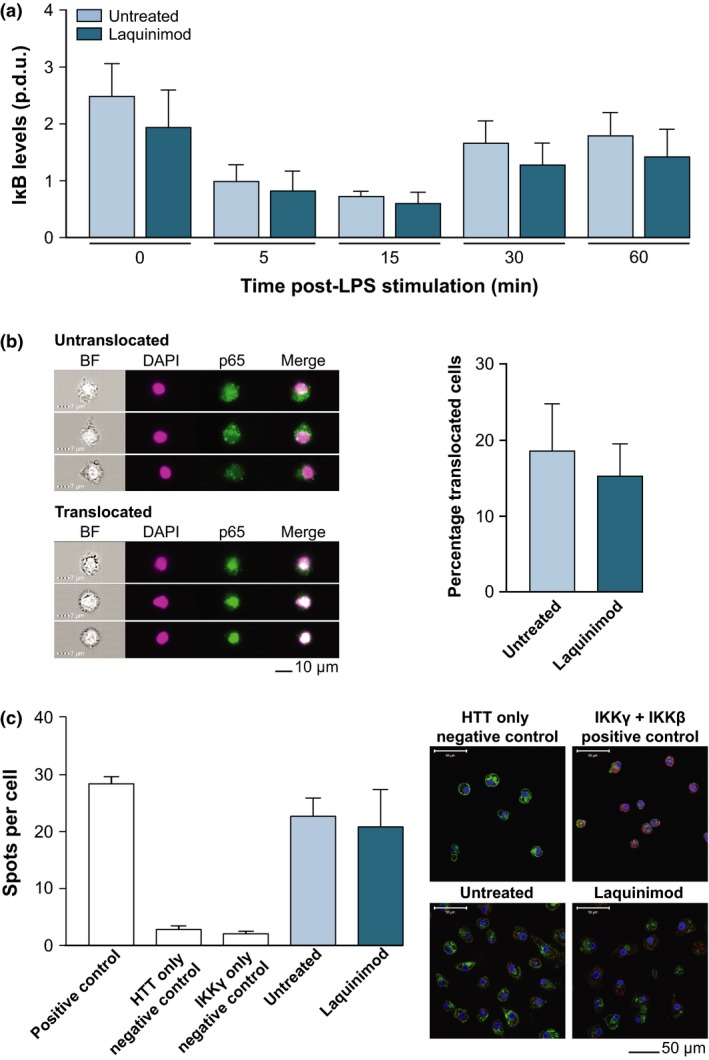
Laquinimod does not affect (a) IκB degradation kinetics, (b) p65 translocation or (c) IKKγ‐mHTT interaction in HD patient myeloid cells. (a) IκB degradation kinetics in lipopolysaccharide (LPS)‐stimulated HD monocytes, with and without 24hPT with 5 μM laquinimod. IκB levels measured using western blotting of cell lysates harvested at 0, 5, 15, 30 and 60 min post‐LPS stimulation. Blots quantified using densitometry giving a procedure defined unit (p.d.u.) for comparison of conditions. Columns = mean average p.d.u. of four biological repeats; error bars = SEM. (b) Nuclear translocation of NFκB transcription factor p65 in manHD monocytes 45 min post‐LPS stimulation, as assessed by imaging flow cytometry. Monocytes received 24hPT with 5 μM laquinimod or untreated. Monocytes were probed for p65 (green) and nuclei were stained with DAPI (pink), as can be seen in example images all taken from the same untreated sample, and percentage translocated cells assessed. Columns = mean average percentage translocated cells from seven biological repeats; error bars = SEM. (c) Interaction between HTT and IKKγ in HD macrophages with or without 24 h 5 μM laquinimod treatment, as assessed by proximity ligation assay (PLA). Cells imaged using fluorescence confocal microscopy. A direct interaction between the two proteins is presented as a fluorescent red spot, nuclei stained with DAPI (blue) and membranes withwheat germ agglutinin (WGA) (green), as can be seen in example images all taken from the same patient cells. Positive and negative controls indicate success of the assay. Columns = mean average spots per cell (untreated condition from 6 biological repeats, laquinimod condition from five biological repeats; five matched pairs); error bars = SEM.

To determine whether laquinimod has the potential to improve NFκB dysfunction in HD, the effect of the compound on IκB degradation kinetics was assessed in HD monocytes. IκB binds NFκB transcription factor p65 in the cytoplasm, preventing its nuclear translocation. Degradation of IκB, however, liberates p65, which then translocates into the nucleus to modulate transcription (Hayden and Ghosh 2012). Consequently, monitoring IκB kinetics is an indirect measure for monitoring p65 nuclear translocation; decreased IκB levels indicates increased nuclear translocation of p65. ManHD monocytes were stimulated with LPS, with or without 24hPT with 5 μM laquinimod, and IκB levels were measured by western blotting at 0, 5, 15, 30 and 60 min post‐stimulation. LPS stimulation resulted in the usual degradation pattern of IκB for HD monocytes (Träger *et al*. 2014), and laquinimod pre‐treatment did not significantly alter (*p* = 0.566) IκB levels from the untreated condition over time post‐LPS stimulation (Fig. 4a). As IκB degradation kinetics were not affected by laquinimod treatment, this suggests that laquinimod may not alter nuclear translocation of the NFκB transcription factor p65 in monocytes. However, because of the indirect method of measuring p65 translocation used, it was necessary to directly monitor nuclear translocation using an alternative method before ruling out an effect of laquinimod in this mechanism.

Imaging flow cytometry was implemented to directly assess p65 nuclear translocation. ManHD monocytes received 24hPT with 5 μM laquinimod, or untreated, for 24 h before 45 min LPS stimulation. Monocytes were labelled with anti‐p65 antibody and DAPI nuclear stain, and run through ImageStream to detect intra/extra‐nuclear localization of p65. NFκB translocation has previously been shown to be pathologically enhanced in HD patient myeloid cells, leading to a hyper‐reactive response to LPS stimulation and increased release of proinflammatory cytokines (Träger *et al*. 2014). 24hPT with 5 μM laquinimod did not significantly alter (*p* = 0.466) the extent of p65 translocation compared to untreated in HD monocytes (Fig. 4b).

It has previously been shown in HD myeloid cells that mHTT directly interacts with the γ subunit of IKK, and it is this dysfunctional interaction which may lead to the observed increase in IκB degradation (Träger *et al*. 2014). PLA was conducted to assess the interaction between mHTT and IKKγ in HD macrophages after 24hPT with 5 μM laquinimod, or untreated. Laquinimod did not significantly alter (*p* = 0.552) the number of observed positive HTT‐IKKγ interactions (spots per cell) compared to untreated, suggesting that laquinimod does not affect the interaction between mHTT and IKKγ in HD macrophages (Fig. 4c).

## Discussion

Systemic levels of proinflammatory cytokines in HD gene carriers are elevated many years before disease manifestation, and monocytes and macrophages are the likely source (Björkqvist *et al*. 2008). HD patient monocytes produce higher levels of cytokines than HV monocytes when stimulated *ex vivo* (Björkqvist *et al*. 2008) and are impaired in their ability to migrate towards chemoattractant stimuli (Kwan *et al*. 2012b). There is evidence that systemic inflammatory processes activate CNS microglia leading to exacerbation of neurodegeneration pathology (Cunningham *et al*. 2007; Perry 2007; Perry *et al*. 2007; Palin *et al*. 2008), and peripheral immune challenges have been shown to exacerbate disease progression in mouse models of HD (Hsiao *et al*. 2013). Therefore, peripheral immune events may directly influence neuroinflammation and neuropathology in HD.

Laquinimod appears to alter hyperactivity of the peripheral immune system in HD as shown by its reduction in cytokine release from HD patient blood‐derived monocytes. In non‐stimulated conditions, laquinimod showed significant effects on HV cytokines but not preHD or manHD cytokine levels. In stimulated monocytes, laquinimod had an overall dampening effect on cytokine release; however, these changes were variable depending on the cytokine, subject group, and laquinimod pre‐treatment conditions. Laquinimod pre‐treatment reduced the release of nearly all cytokines from stimulated preHD and/or manHD monocytes compared to the untreated condition. This effect was most consistent following 24hPT, and interestingly had an effect of larger magnitude on HD monocytes than on HV monocytes, where the changes (if any) in the HV group were relatively minor. In particular, our data provides evidence for this for the Th1‐activating proinflammatory cytokines IL‐1β, IL‐8 and TNFα and the Th2‐activating cytokines IL‐5, IL‐13 and anti‐inflammatory IL‐10. It is important to note that the natural differences in cytokine release from primary human monocytes (HV and HD) leads to large variations in cytokine level measurements, which introduces some limitations to this system. Consequently, it is not always possible to determine whether lack of a statistically significant effect is because of there being no effect or because of random variation in measurements masking an effect that may be present. Overall, the sample size limits the precision for which between condition and between subject group differences can be assessed. However, there is evidence that laquinimod does have an effect on cytokine release from HD monocytes, and that this effect may be different than the effect of laquinimod on HV monocytes.

Cytokine level trends across laquinimod treatment conditions are generally consistent in terms of laquinimod‐induced changes being greater in magnitude at the 5 μM drug concentration and a longer pre‐treatment duration. However, this did not usually extend to the 24hPT+24hCT condition, where in many cases a much smaller change from the untreated condition occurred than would have been predicted, or there was even a change in the opposite direction. Indeed, with the exception of IL‐10, there were small increases in the Th2‐activating cytokines in the 24hPT+24hCT condition. Thus, it is important to note that the 24hPT+24hCT condition may need to be considered independently from the pre‐treatment only conditions, as laquinimod was present during stimulation. It is possible that an alternative mechanism of action is at play under these conditions, such as competitive binding or confounding cross talk between intracellular signalling pathways.

We did not observe an overall shift in cytokine balance from Th1‐ to Th2‐activating cytokine release in HD monocytes, which has been reported previously in MS (Zou *et al*. 2002; Yang *et al*. 2004; Brück and Wegner 2011; Schulze‐Topphoff *et al*. 2012). The shift in cytokine balance previously reported was identified in systems quite different than those used here. Zou *et al*. (2002) and Yang *et al*. (2004) both describe a decrease in proinflammatory cytokines along with an increase in Th2‐activating and anti‐inflammatory cytokines *in vivo* in rat models of MS following 15 days of daily laquinimod administration. Zou *et al*. found a decrease in the number of IFNγ‐ and TNFα‐expressing cells, with an increase in the number of IL‐4‐expressing cells in sciatic nerve sections, but in mononuclear cells (MNCs) isolated from lymph node they only looked at release of the proinflammatory cytokines, which decreased. Yang *et al*. describe an increase in the number of spleen MNCs expressing mRNA for the Th2‐activating cytokines IL‐4, TGFβ1 and IL‐10 and a decrease in the number of cells expressing mRNA for proinflammatory IL‐12 and TNFα, however, in PBMCs they report a decrease in the number of cells expressing TNFα mRNA with no change in the other cytokines. Schulze‐Topphoff *et al*. (2012) also report their findings in an *in vivo* animal model of MS, describing a laquinimod‐induced cytokine shift in cells isolated from spleen. Brück and Wegner (2011) report a Th1‐ to Th2‐activating shift following daily laquinimod administration in a MS mouse model, but in PBMCs isolated from HVs they only describe decreases in proinflammatory cytokines. In our model, we push isolated PBMCs towards an M1 phenotype by stimulating them with LPS and IFNγ. In this setting an increase in Th2‐activating cytokines was unlikely to be observed, however, laquinimod pre‐treatment did reduce overall cytokine production, including those which promote Th1 activation, suggesting that the functional signatures of laquinimod‐treated HD monocytes are somewhere between the M1 and M2 polarization extremes following stimulation (Martinez and Gordon 2014). Overall, our evidence for a laquinimod‐induced decrease in cytokine release from human HD monocytes is supported by the current literature on laquinimod effects in MS models. Our findings for lack of a Th1‐ to Th2‐activating shift in cytokine balance is also consistent with the effects seen following laquinimod pre‐treatment of LPS‐activated human microglial cultures, where laquinimod significantly reduced the secretion of the proinflammatory cytokines TNFα, IL‐1β, IL‐12p70 and IL‐6, and the anti‐inflammatory/regulatory cytokines IL‐4, IL‐10 and IL‐1ra (Mishra *et al*. 2014).

In the Björkqvist *et al*. (2008) study comparing peripheral cytokine levels in the plasma of HV and HD patient subgroups, there were increased levels of both Th1‐ and Th2‐activating cytokines in HD compared to HV, and these increases correlated with disease progression. The Th1‐activating cytokines IL‐6, IL‐8 and TNFα, and the Th2‐activating cytokines IL‐4 and anti‐inflammatory IL‐10 were elevated in HD patient plasma compared to HV plasma. A combination of IL‐6, IL‐8 and IL‐10 cytokine increases was positively correlated with disease progression from preHD through to manHD, and could also be used to discriminate HV from HD gene carriers (both pre‐manifest and manifest). In this study, laquinimod shows dampening of the hyper‐reactive release of cytokines by HD monocytes, and this may be a way of reducing the elevated plasma cytokine levels observed in HD patients and the increasing levels in advancing disease, both peripherally and in the brain.

It may seem counterintuitive to reduce potent proinflammatory factors such as IL‐1β and TNFα whilst additionally decreasing the key anti‐inflammatory cytokine IL‐10. However, IL‐10 levels, which are shown to be high in HD patients, are also abnormally elevated in Alzheimer's disease (AD) patient brains (Guillot‐Sestier *et al*. 2015), and IL‐10 has recently been highlighted as harmful in mouse models of AD; increasing brain amyloid‐β (Aβ) accumulation, exacerbating memory impairment and suppressing microglial Aβ phagocytosis (Chakrabarty *et al*. 2015). As HD and AD share similar pathologies of protein aggregation and neurodegeneration, and are both diseases exacerbated by dysregulated inflammation, it may be that the laquinimod‐induced reduction in IL‐10 from HD peripheral myeloid cells is beneficial, and may contribute to resolution of the innate immune system cytokine balance.

Previous studies on laquinimod mechanism of action present evidence for a role of the NFκB pathway in astrocytes. Laquinimod reduced astrocytic NFκB activation in cuprizone‐treated mice and primary human astrocyte cultures (Brück *et al*. 2012), and gene expression microarrays on laquinimod‐treated PBMCs isolated from MS patients revealed down‐regulation of key genes downstream of NFκB signalling (Gurevich *et al*. 2010). The results of this study, however, do not support a role for laquinimod mechanism of action downstream of NFκB activation in primary human HD monocytes. Laquinimod did not affect IκB degradation kinetics, nuclear translocation of p65 nor IKKγ‐mHTT protein–protein interactions, from the untreated condition. This is in accordance with work by Brück *et al*. who found that NFκB activation was markedly reduced by laquinimod in astrocytes, but not in microglia, the resident myeloid cells of the CNS, in stimulated primary mouse cultures (Brück *et al*. 2012).

In summary, 5 μM laquinimod applied to HD patient peripheral myeloid cells for 24 h appears to reduce hyper‐reactive cytokine production in response to LPS stimulation. This is true for Th1‐activating proinflammatory cytokines and Th2‐activating cytokines including IL‐10. Altering peripheral immune responses modulates central pathology and disease progression in HD, hence laquinimod is a promising candidate for dampening the harmful effects of a dysfunctional innate immune system in HD. The mechanism for this does not appear to be downstream of NFκB activation in these cells. Laquinimod mechanism of action is currently under investigation.

## Conflicts of interest

Laquinimod is produced by Teva Pharmaceutical Industries Ltd, Israel. LH, MRH and PL are employees of Teva. LD has received travel expenses for attending meetings and SJT has received financial grant support for research from Teva Pharmaceutical Industries Ltd, Israel.

## Supporting information


**Figures S1 and S2.** Primary non‐stimulated human monocyte cultures from HV, preHD and manHD received 2hPT or 24hPT with 1 or 5 μM laquinimod, or 24hPT+24hCT with 5 μM laquinimod. Levels of cytokine release were measured in culture supernatants and normalized to cell culture protein. Supplementary figure 1 displays results for the Th1‐activating cytokines and Figure S2 displays results for IL‐2 and the Th2‐activating cytokines measured. Columns = mean average log cytokine level of biological repeats; error bars = SEM; *p<0.05, **p<0.01.
