# Supplementary material for: Laquinimod dampens hyperactive cytokine production in Huntington's disease patient myeloid cells
Source: J Neurochem. 2016 Apr 5;137(5):782–94. doi: 10.1111/jnc.13553 (PMC4982105; doi:10.1111/jnc.13553)

Supplementary

Title: Laquinimod dampens hyperactive cytokine production in Huntington's disease patient myeloid cells

Authors: Lucianne Dobson[1], Ulrike Träger[1,5], Ruth Farmer[2], Liat Hayardeny[3], Pippa Loupe[3], Blair R Leavitt[4], Michael R Hayden[3], and Sarah J Tabrizi[1].

Author affiliations:

1. University College London, Institute of Neurology and National Hospital for Neurology and Neurosurgery, Department of Neurodegenerative Diseases, London, UK
2. London School of Hygiene & Tropical Medicine, Department of Medical Statistics, London, UK
3. Teva Pharmaceuticals, Research and Development, Netanya, Israel.
4. The University of British Colombia, Centre for Molecular Medicine and Therapeutics, Vancouver, Canada
5. Now at German Cancer Research Centre, Immune Tolerance, Tumour Immunology Program, Heidelberg, Germany

Supplementary figures 1 and 2 legend: *Primary non-stimulated human monocyte cultures from HV, preHD and manHD received 2hPT or 24hPT with 1 or 5 µM laquinimod, or 24hPT+24hCT with 5 µM laquinimod. Levels of cytokine release were measured in culture supernatants and normalised to cell culture protein. Supplementary figure 1 displays results for the Th1-activating cytokines and supplementary figure 2 displays results for IL-2 and the Th2-activating cytokines measured. Columns = mean average log cytokine level of biological repeats; error bars = SEM; *p<0.05, **p<0.01.*

SUPPLEMENTARY FIGURE 1: Th1-activating cytokine levels in non-stimulated monocytes pre-treated with laquinimod

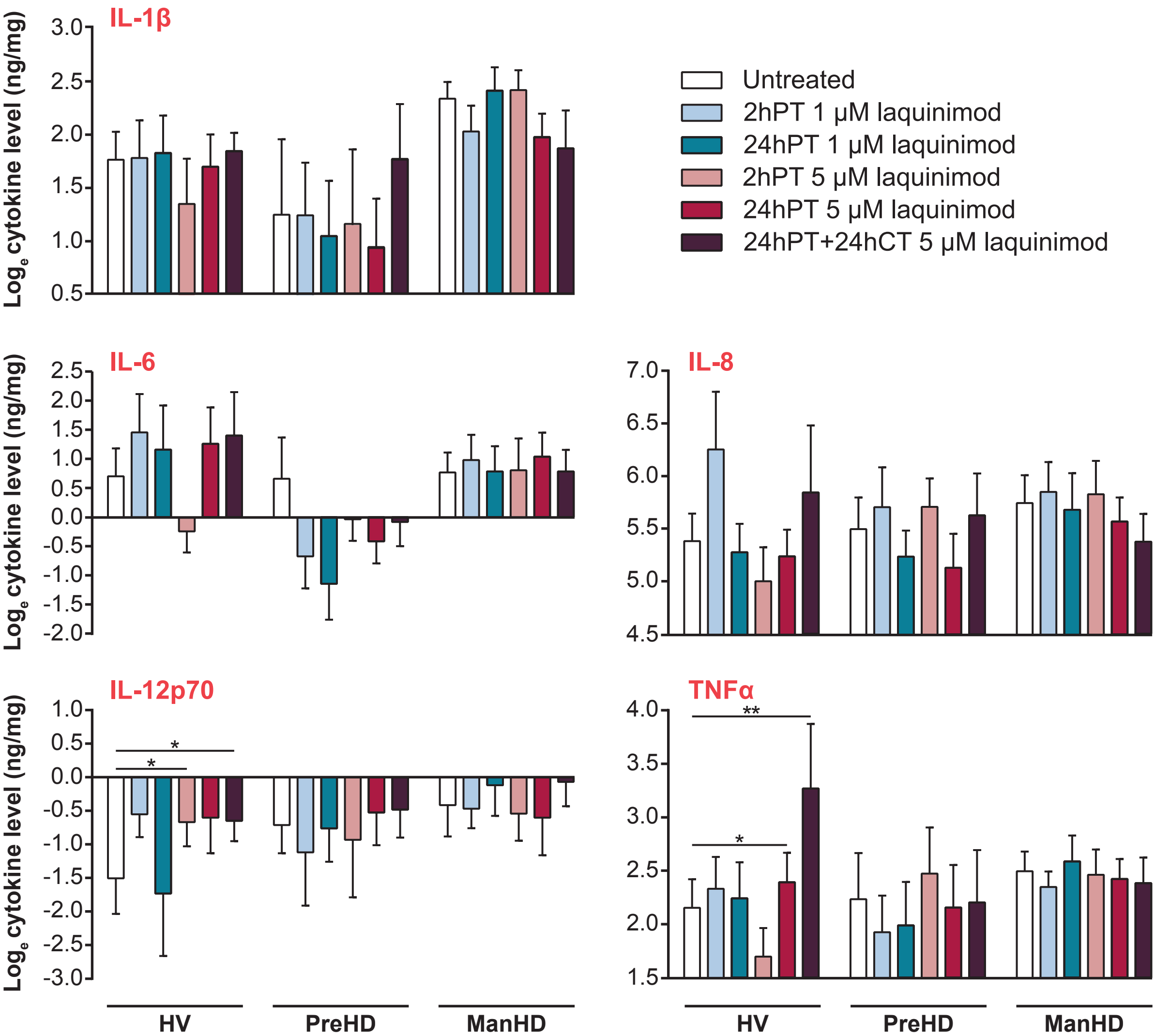

SUPPLEMENTARY FIGURE 2 IL-2 and Th2-activating cytokine levels in non-stimulated monocytes pre-treated with laquinimod

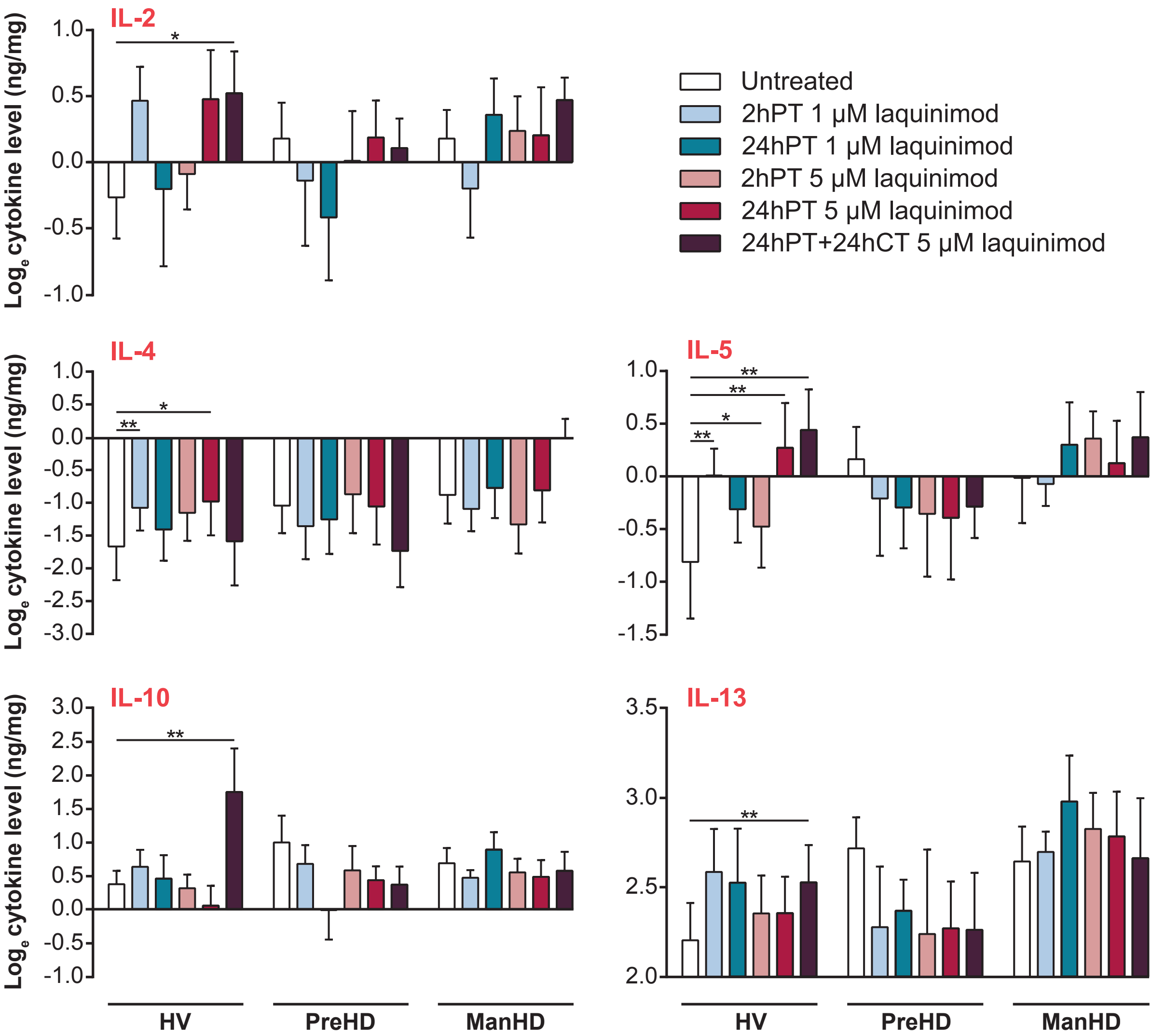

Supplement: Supplementary file 1 — Figures S1 and S2. Primary non‐stimulated human monocyte cultures from HV, preHD and manHD received 2hPT or 24hPT with 1 or 5 μM laquinimod, or 24hPT+24hCT with 5 μM laquinimod. Levels of cytokine release were measured in culture supernatants and normalized to cell culture protein. Supplementary figure 1 displays results for the Th1‐activating cytokines and Figure S2 displays results for IL‐2 and the Th2‐activating cytokines measured. Columns = mean average log cytokine level of biological repeats; error bars = SEM; *p<0.05, **p<0.01. [file JNC-137-782-s001.pdf]
